# Pregnancy in a Woman with Latent Myeloproliferative Neoplasm Induced Chronic Portal Vein Thrombosis, Portal Cavernoma, and Gastric Varices

**DOI:** 10.1155/2019/5702983

**Published:** 2019-03-14

**Authors:** Andréanne Durivage, Geneviève Le Templier, Annabelle Cumyn, Nadine Sauvé

**Affiliations:** Department of General Internal Medicine, Faculty of Medicine, University of Sherbrooke, Quebec, Canada

## Abstract

Extra-hepatic portal vein thrombosis (EHPVO) represents the obstruction of the portal vein outside the liver and is not related to chronic liver disease or neoplasia. In chronic EHPVO, collateral veins and portal hypertension develop, resulting in splenomegaly and variceal formation. Myeloproliferative neoplasms (MPN) are the most frequent acquired etiology of EHPVO. These conditions put pregnant women at increased risk of vascular complications, including venous thrombosis, occlusion of the placental circulation, and variceal bleeding. In this report, we present a 36-year-old pregnant woman with chronic, anticoagulated EHPVO secondary to latent MPN who developed severe intrauterine growth restriction and had cesarean section at 32+1 weeks for increased umbilical doppler resistance and breech presentation. The article will emphasize outcome and management of pregnancies complicated by chronic EHPVO, portal hypertension, and MPN.

## 1. Introduction

Extra-hepatic portal vein thrombosis (EHPVO), also named noncirrhotic portal vein thrombosis (PVT), represents obstruction of the extra-hepatic portal vein unrelated to chronic liver disease or neoplasia [1]. Portal venous obstruction is caused by both thrombotic (acquired or hereditary) and nonthrombotic causes. The acquired conditions include abdominal inflammation, infections, surgery, obesity, oral contraceptive intake, pregnancy, postpartum period, and myeloproliferative neoplasms (MPN), which is the most frequent [1, 2].* Valla et al* reported an incidence of 58% of latent MPN in western patients with EHPVO of unknown etiology. Among those patients, 57% developed an overt MPN during followup [3]. On the other hand, very few PVT are diagnosed throughout pregnancy or postpartum, representing less than 4% of PVT cases in the latest series [4].

In the absence of recanalization, a network of collateral veins develops as a compensatory mechanism of venous rescue to bypass the obstruction [2, 5]. These porto-portal collaterals are given the name “portal cavernoma” [6]. They begin to form rapidly in the first 6 to 20 days following acute thrombosis and are complete 3 to 5 weeks thereafter [2]. Thus, the presence of collateral veins on imaging and signs of portal hypertension, such as splenomegaly and varices, confirm the diagnosis of chronic EHPVO [7]. Hypersplenism and variceal bleeding are frequent manifestations of chronic PVT [6]. Splenomegaly, ranging from moderate to massive, is always present and can be a presenting feature [1]. Furthermore, 30-40% of patients with chronic PVT develop gastric varices secondarily to portal hypertension [6]. There is a constant risk of gastrointestinal bleeding associated with portal cavernoma, sometimes fatal [5].

Women of childbearing age represent nearly 25% of patients with noncirrhotic PVT [5, 6]. Because liver function is usually maintained, their fertility is considered normal [4, 6]. It is well established that pregnancy is associated with an increased risk of thrombosis, a risk that is roughly six times higher than in nonpregnant women [8]. MPN also amplifies thrombotic risk, making pregnant woman with this condition at higher risk of vascular complications including venous thromboembolism and occlusion of the placental circulation [8]. With that in mind, we present a 36-year-old woman with chronically anticoagulated PVT secondary to latent myeloproliferative neoplasm who was referred to our clinic for preconception counselling. The report will emphasize on outcome and management of pregnancies complicated by chronic PVT, portal hypertension, and MPN.

## 2. Case Report

A 36-year-old gravida 1 para 0 presented to the obstetric medicine clinic for preconception counselling. Her medical history was notable for chronic thrombosis of the extra-hepatic portal, splenic, and mesenteric veins since 2011. She had developed cavernous transformation in her portal vein, portal hypertension, splenomegaly, and secondary grade 1-2 gastric varices. A few years after the thrombosis was discovered, a diagnosis of latent myeloproliferative neoplasm was made on the basis of a positive Jak-2 mutation (V617F, 1.65% mutational allele burden) and increased cellularity on the bone marrow biopsy. Until now, she has never developed polycythemia, thrombocytosis, or leukocytosis (last hemoglobin level 135 g/L, platelet count 349×10^9^/L, and leukocytes count 8.8×10^9^/L). The patient was also known for essential hypertension, hypothyroidism, migraine, and obesity. Her first pregnancy (2011), while on depo-provera and warfarin, was interrupted voluntarily. In 2012, she had bilateral salpingectomy with unilateral left ovariectomy for possible endometriosis.

On medication review, she had taken warfarin after the thrombosis diagnosis until it was replaced by dalteparin (18 000 units once a day, subcutaneous injection) because of difficulty to reach and maintain the target international normalized ratio (INR) despite doses greater than 20 mg daily. The patient decided to stop dalteparin nine months after the initial thrombosis. It was not replaced by another anticoagulant because of the risk of variceal bleeding. However, aspirin 80 mg once daily was prescribed to address established platelets, leukocytes, and endothelium interaction in the pathogenesis of vascular occlusion in MPN [8, 9]. Aspirin was replaced by clopidogrel 75 mg once daily after she developed an allergic reaction. Rivaroxaban 20 mg once daily was finally added to her medication when splenic infarctions were discovered at the time of an episode of abdominal pain. At her preconception visit, she was taking rivaroxaban 20 mg once daily and clopidogrel 75 mg once daily.

After a frank and open counselling about the risks associated with a pregnancy, namely, thrombosis (especially considering the* in vitro* fertilization and MPN) and variceal bleeding, she decided to begin the process of* in vitro* fertilization. Rivaroxaban was replaced by once a day subcutaneous therapeutic dose of dalteparin (18 000 units) and clopidogrel was stopped. She was already taking nadolol 20 mg once daily for her gastric varices.

At her 5th week of pregnancy, she had vaginal bleeding, but after a few days of bed rest, it did not recur. Her ultrasound at gestational week 22 showed fetal growth at the 10th percentile. She was hospitalized for 48h at 26 weeks of pregnancy for severe intrauterine growth restriction (IUGR) secondary to placental insufficiency. Nadolol may have also contributed to the IUGR [10]. There was no preeclampsia so she was discharged from the hospital. She finally had a cesarean section at 32+1 weeks for severe IUGR and increased umbilical doppler resistance. This delivery route was chosen because of breech presentation.

At her 3 weeks postpartum followup, she confirmed her decision not to pursue breastfeeding. After a long discussion, it was then decided to stop dalteparin and start apixaban 2.5 mg twice daily, a reduced dose to minimize the risk of variceal bleeding while preventing another thrombosis.

## 3. Discussion

Outcomes of such cases are uncommon in the medical literature. In the last decade, a total of 83 pregnancies in 43 patients with PVT and portal hypertension were reported in 3 studies [5, 7, 11]. Firstly in 2008, a research reported 12 pregnancies in 5 patients with noncirrhotic portal hypertension [11]. Only one woman had hematemesis at 8 weeks of gestation and was successfully managed with blood transfusion and endoscopic sclerotherapy. In this series, the incidence of small for gestational age babies was increased (44%) [11]. The second study in 2011 reported 26 pregnancies in 14 women with EHPVO [7]. None had variceal bleeding during pregnancy. Anemia and thrombocytopenia were the most common complications caused by hypersplenism. Authors concluded that outcomes are expected to be good if the disease is diagnosed and adequately treated before conception [7]. Finally, in 2012,* Hoekstra et al.* reported 45 pregnancies in 24 patients with PVT [5]. Three patients had esophageal varices bleeding during pregnancy, all without adequate prophylaxis. Postpartum bleeding occurred in 4 women, with only one being anticoagulated at that time. No difference was appreciated in bleeding rate whether patients were anticoagulated or not. There was an association between higher platelet count at diagnosis of PVT and unfavorable outcome during pregnancy. HELLP (hemolysis, elevated liver enzymes, low platelet count) syndrome complicated 2 pregnancies, both presenting infarcts on placental inspection. One of these women had underlying MPN, suggesting this prothrombotic disorder may cause unfavorable pregnancy outcome by occluding placental vessels [5].

Direct oral anticoagulants (DOAC) are sometimes used “off-label” for the treatment of PVT [12]. These medications offer some advantages over vitamin K antagonists, specifically the stable dose without the necessity for laboratory monitoring [13]. Despite the paucity of data on the use of DOACs in the treatment of venous thrombosis in atypical locations, their use is expanding in splanchnic venous thrombosis (SVT) [12, 13].* De Gottardi et al. *presented the largest series on DOACs in patients with SVT. In this retrospective study, among the 58 patients without cirrhosis, 38 (65%) were anticoagulated for PVT. Adverse events happened in five patients (8,6%), 2 thrombotic and 3 hemorrhagic complications, leading to the change of anticoagulant in 4 cases [12]. Even if DOACs seem to be safe and effective in SVT, further data are needed [12, 13].

What about the risk of variceal bleeding in pregnancy? There are two perspectives on the matter. Firstly, some authors think that variceal bleeding increases with pregnancy [1, 6, 10, 14]. Even in women with no prior history of variceal bleeding, the gravid state can sometimes trigger a first episode [1]. This feared complication can happen at all stages of pregnancy but is more frequent in second and third trimesters and during the second stage of labor [10]. The increased risk of bleeding comes from the physiological hemodynamic changes of pregnancy, which bring a hyperdynamic and hypervolemic state and thus increase the inflow to the collaterals [6, 10, 14]. Conversely,* Sumana et al* and* Kochhar et al* hypothesize that even if cardiac output and blood volume increase during pregnancy, the splanchnic blood flow and the portal pressure remained stable because the majority of the additional blood circulates through the uterine arteries to the fetus [11, 15]. Indeed, variceal bleeding during pregnancy is probably less frequent than initially thought [11].

When the woman has a history of variceal bleeding, it is important to treat the varices before conception, either with endoscopic variceal ligation or sclerotherapy, since the incidence of upper gastrointestinal bleeding decreases with prepregancy eradication of varices [1, 6, 7, 11, 16]. Even in the absence of prior variceal bleeding, primary prophylaxis should be given to pregnant woman at risk [6, 10]. There is not enough data to decide whether beta-blockers or endoscopic therapy should be favored [1, 17]. Nonselective beta-blockers are considered safe in pregnancy, though nadolol and propranolol may induce growth retardation, fetal bradycardia, and neonatal hypoglycemia [10].

Lastly, delivery route should be chosen according to standard indications [1, 11]. Vaginal delivery is preferred with adequate analgesia and assisted second stage of labor to limit straining effort [4, 6, 10, 11]. Cesarean section should be reserved for usual obstetric reasons [1, 11]. Risks specific to caesarean delivery must be considered in women with portal hypertension, including surgical bleeding from thrombocytopenia or direct injury to abdominal wall varices and postoperative ascitic decompensation [4, 6]. Moreover, caesarean operation augments the chance of postpartum venous thromboembolism, a risk that is already high in women with PVT [4].

## 4. Conclusion

Although there will always be a risk of variceal bleeding during pregnancy in woman with PVT and portal hypertension, outcome is generally good in the presence of preconception evaluation and primary or secondary prophylaxis for varices with beta-blockers and endoscopic therapy. Available data permit us to propose that pregnancy is not contraindicated in these patients when the disease is treated and stable. Anticoagulation should be continued to prevent new thrombosis. Pregnant women at risk should be followed in a center where expertise and multidisciplinary team are available.
